# Cincinnati Medical Society

**Published:** 1835

**Authors:** 


### IX. Cincinnati Medical Society.

The fifth annual election of the Cincinnati Medical Society, on the 4th of March, resulted in the choice of the following officers for 1835:

Dr. L. C. Rives, President.
Dr. C. R. Cooper, 1st Vice President.
Dr. J. M. Mason, 2d Vice President.
Dr. W. Wood, Chairman.
Dr. B. F. Williams, Secretary.
Dr. J. T. Shotwell, Treasurer.
Dr. I. S. Dodge, Librarian.
Dr. J. Colby, Botanist.
Dr. J. R. Ridell. Mineralogist.
Drs. D. Drake, C. Woodward, S. Reed, Library Committee.
Drs. A. Judkins, M. Flagg, S. Bonner, Vaccine Committee.

The Cincinnati Medical Society is the only association of the kind in existence in Cincinnati. It is incorporated. The object for which it was instituted is professional improvement.

The society respectfully solicits from the physicians of the Valley of the Mississippi, specimens of our indigenous medical plants, particularly those found in remote and comparatively unexplored places; it is also desirous of receiving specimens of our native minerals. Anxious to make its library a depository of rare and curious books, it will thankfully receive any, which detached practitioners may be willing to part with. Its collection has recently been enlarged by the library of the late First District Medical Society.

Members of the profession can at all times throughout the year, be supplied with fresh and genuine vaccine infection, free of charge, by addressing a letter, post paid, to either of the members of the Vaccine Committee.

